# A New Approach for Controlling Mesoporosity in Activated Carbon by the Consecutive Process of Air Oxidation, Thermal Destruction of Surface Functional Groups, and Carbon Activation (the OTA Method)

**DOI:** 10.3390/molecules26092758

**Published:** 2021-05-07

**Authors:** Panuwat Lawtae, Chaiyot Tangsathitkulchai

**Affiliations:** School of Chemical Engineering, Institute of Engineering, Suranaree University of Technology, Muang District, Nakhon Ratchasima 30000, Thailand; D6040246@g.sut.ac.th

**Keywords:** activated carbon, longan seed, mesoporosity, pore size distribution, physical activation

## Abstract

A new and simple method, based entirely on a physical approach, was proposed to produce activated carbon from longan fruit seed with controlled mesoporosity. This method, referred to as the OTA, consisted of three consecutive steps of (1) air oxidation of initial microporous activated carbon of about 30% char burn-off to introduce oxygen surface functional groups, (2) the thermal destruction of the functional groups by heating the oxidized carbon in a nitrogen atmosphere at a high temperature to increase the surface reactivity due to increased surface defects by bond disruption, and (3) the final reactivation of the resulting carbon in carbon dioxide. The formation of mesopores was achieved through the enlargement of the original micropores after heat treatment via the CO_2_ gasification, and at the same time new micropores were also produced, resulting in a larger increase in the percentage of mesopore volume and the total specific surface area, in comparison with the production of activated carbon by the conventional two-step activation method using the same activation time and temperature. For the activation temperatures of 850 and 900 °C and the activation time of up to 240 min, it was found that the porous properties of activated carbon increased with the increase in activation time and temperature for both preparation methods. A maximum volume of mesopores of 0.474 cm^3^/g, which accounts for 44.1% of the total pore volume, and a maximum BET surface area of 1773 m^2^/g was achieved using three cycles of the OTA method at the activation temperature of 850 °C and 60 min activation time for each preparation cycle. The two-step activation method yielded activated carbon with a maximum mesopore volume of 0.270 cm^3^/g (33.0% of total pore volume) and surface area of 1499 m^2^/g when the activation temperature of 900 °C and a comparable activation time of 240 min were employed. Production of activated carbon by the OTA method is superior to the two-step activation method for better and more precise control of mesopore development.

## 1. Introduction

Activated carbon is one of the most versatile porous adsorbents, and is used in many separations and purification processes for both gas and liquid phase systems [1]. Activated carbon is an amorphous carbon-based material which exhibits a high degree of porosity, an extended surface area, microporous structure, high adsorption capacity as well as a high degree of surface reactivity. Typically, activated carbon can be synthesized from a variety of low-cost cellulosic materials such as wood, rice straw, nutshell, corn hull, coconut shell, oil-palm shell, longan seed, bamboo and peach stone, or carbonaceous materials such as bituminous coal, lignite, and peat [2,3]. It can be prepared through a two-step physical activation with oxidizing agents such as carbon dioxide (CO_2_), oxygen (O_2_), or steam [1,4], or a single-step preparation through wet impregnation of a precursor with inorganic chemical agents such as zinc chloride (ZnCl_2_), potassium hydroxide (KOH), sodium hydroxide (NaOH) or phosphoric acid (H_3_PO_4_), followed by a carbonization process [5].

In general, the preparation of activated carbon by physical activation will predominantly produce micropores [4]. This type of carbon is suitable for use in gas adsorption applications. However, effective adsorption in liquids, both in terms of kinetics and adsorption capacity, requires activated carbon with a high proportion of mesopore volume. To be able to use activated carbon in the separation processes more widely and more efficiently, it is necessary to develop a process of activated carbon synthesis that can control proportionally the amounts of micropores and mesopores. The control of mesopores in activated carbon is mostly achieved through chemical activation, which consists of several methods such as a one-step chemical activation [6,7,8,9,10,11,12], a two-step chemical activation [13,14,15,16], a hydrothermal pretreatment followed by a simple chemical activation [17,18], and chemical activation with dual activation agents [19]. Furthermore, the combined chemical and physical activation has also been used to control the amount of mesopores in activated carbon [20,21,22,23,24]. On the other hand, attempts to effectively control mesopore volume in activated carbon by using only a physical activation approach have not yet been reported to date. The use of chemical activating agents may cause a serious environmental problem and their corrosiveness is harmful to process equipment. Hence, this research work aims to study a new method based on the combined processes of oxidation, thermal destruction, and activation, which is purely based on a physical activation method.

Normally, the synthesis of activated carbon by the gasification reaction of a precursor with carbon dioxide mainly produces a large number of micropores [25]. The extent of the reaction, and hence the developed pore structure, is determined by the activation time and temperature. Now, if the course of the activation process is interrupted by air oxidation that creates new surface groups, followed by the destruction of those surface groups by heat treatment at a high temperature in an inert gas, the gasification reactivity of the original micropore surfaces should be enhanced due to the increasing number of unpaired electrons and free radicals resulting from the bond disruption. Therefore, when the activated carbon is reactivated with CO_2_, it is possible that the original micropores with a higher surface reactivity could be enlarged by the gasification reaction, resulting in a greater amount of mesopores. This method was proposed to produce a high ratio of mesopore volume to total pore volume and highly porous activated carbons when compared with the conventional two-step physical activation. This new method is capable of producing the desired amounts of micropores and mesopores by precise control of the conditions used during the various preparation steps. The advantages of this method are that it is easy to operate, has a low cost, and will not be the major cause of any pollution, since the process is conducted solely in the gas phase using only air, inert gas, and carbon dioxide, and it is not associated with any acid solutions or oxidizing agents in the liquid phase.

The objective of this study is therefore to propose a new and simple method for the preparation of mesoporous activated carbon from longan fruit seed by the combined consecutive processes of oxidation, thermal destruction, and activation, abbreviated as the OTA preparation method. The parameters being studied included time and temperature during the activation step, the number of repeated OTA cycles, and the pore characteristics of the starting activated carbons. Furthermore, the Grand Canonical Monte Carlo (GCMC) simulation was employed to determine the pore size distribution of the activated carbon produced from longan seed biomass. The underlying mechanism of pore development by the OTA method was also proposed and the supporting experimental evidence was presented.

## 2. Results and Discussion

### 2.1. Thermal Decomposition Behavior of Longan Seed Precursor

**Figure 1:**
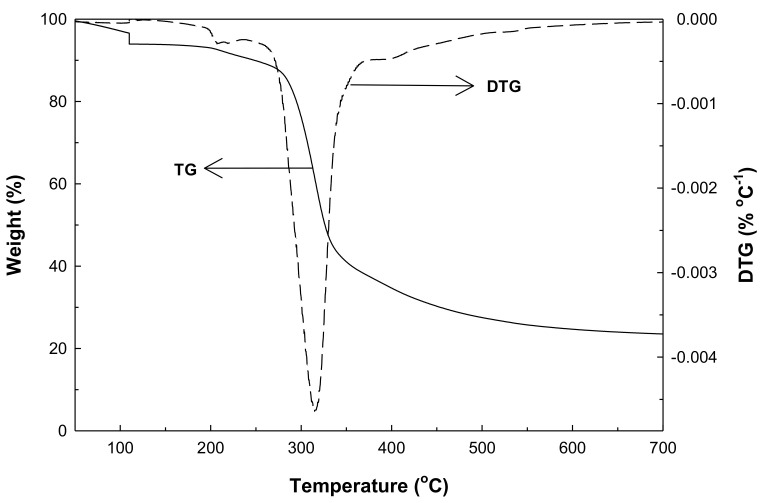
Typical residual weight (TG) and weight loss rate (DTG) for the non-isothermal pyrolysis in the nitrogen atmosphere of longan seed biomass using a thermogravimetric analyzer (TGA).

Figure 1 shows plots of TG and its first derivative (DTG) data for the non-isothermal heating of the longan seed under nitrogen flow in the TGA from room temperature (30 °C) to the final temperature of 700 °C at the heating rate of 10 °C/min to study the thermal decomposition behavior of the logan seed precursor. Weight loss of about 13 wt% was observed when the sample was heated from room temperature to about 200 °C, and this was attributed mainly to the release of equilibrium moisture remaining in the longan seed sample. Two regions of weight loss behavior upon increasing pyrolysis temperature were observed. In the first region, the weight loss due to pyrolysis in N_2_ decreased rapidly over the temperature range from 250 to 400 °C due to the release of most volatile substances caused by the decomposition of cellulose and hemicellulose [26]. The maximum weight loss rate occurred at 320 °C with the main pyrolysis decomposition of longan seed progressing over this temperature range. The second region for the temperature above 400 °C showed a slow decrease in the remaining weight and finally approached a constant yield of solid char of about 25 wt%.

### 2.2. Proximate and Ultimate Analyses of Longan Seed Precursor and Activated Carbon

Table 1 shows the results of proximate analysis of the longan seed used in this study, along with those of other biomass wastes. The fixed carbon content of longan seed from the present work is 22.34 wt%, with a relatively low ash content of 1.15 wt%. Volatile content is high (76.51 wt%), which is typical for biomass materials. These values are comparable with those of the other biomasses reported in the literature. Therefore, with reasonably high carbon content and low ash composition, the longan seed precursor used in this study could be used as a promising precursor for activated carbon production.

Table 2 shows the proximate and ultimate analyses of activated carbons prepared by the two-step activation and the OTA method. For the two-step activation method, increasing the activation time from 60 to 240 min decreased the fixed carbon content by about 4.2% from 86.25 to 82.58 wt%. Obviously, the reduction in fixed carbon is the result of carbon removal by the CO_2_ gasification during the activation step. The volatile content also decreased with the increase in activation time, and this is possibly caused by the additional devolatilization of char at the high activation temperature of 850 °C. The ultimate analysis also showed a decrease in carbon content as the activation time was increased from 60 to 240 min, in line with the results of the proximate analysis. Hydrogen content increased by about 20%, while the oxygen content increased much more, by about 300%.

Similar to the two-step activation, the fixed carbon content of activated carbon produced by the OTA method decreased with each treatment cycle (60 min activation time for each cycle). Volatile content also slightly decreased for activated carbons from cycle 1 to cycle 3. It is interesting to note that for the same activation time, the percentage fixed carbon produced by the OTA method was lower than that of the two-step activation method when, for example, comparing A850-180 vs. A850-60-2 and A850-240 vs. A850-60-3. This proves that the gasification reaction of carbon from the OTA method is faster (more reactive) than that of carbon prepared by the two-step activation.

### 2.3. N_2_ Isotherms of Prepared Activated Carbons

**Figure 2:**
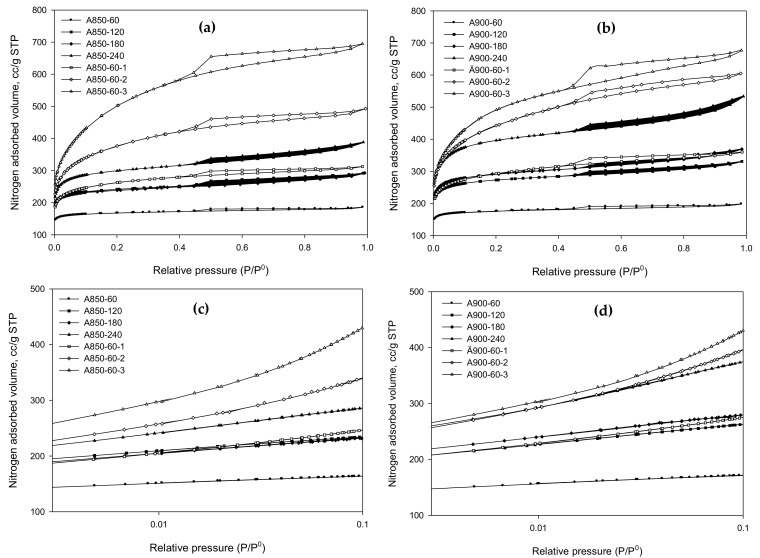
N_2_ adsorption isotherms of microporous activated carbons prepared by the two-step activation method and mesoporous activated carbons prepared by the OTA method at two activation temperatures of (**a**) 850 °C and (**b**) 900 °C, while (**c**,**d**) show the corresponding isotherms at a low-pressure range (*p*/*p*^0^ < 0.1) on a semi-log scale.

The isotherms of N_2_ adsorption by the prepared activated carbons are presented in Figure 2 for the activated carbon series A850 and A900. The variation of isotherms over a low-pressure range of relative pressure less than 0.1 is also demonstrated on a semi-log scale, as shown in Figure 2c,d. It appeared that the effect of pressure on the adsorption isotherms is the same for both the low and high relative pressures. The activated carbons A850-60 and A900-60, prepared by the two-step activation with CO_2_, showed Type I isotherm according to the IUPAC classification [29], typified by a sharp increase in the amount of N_2_ adsorbed at low pressures and followed by a long plateau region at higher pressures. This adsorption behavior is indicative of adsorption in micropores either in pores of molecular dimensions by the pore-filling mechanism at very low pressures or in larger micropores over a range of higher pressures [30]. It appeared that as the activation time was progressively increased from 60 to 240 min, the amounts of N_2_ adsorbed tended to increase, due largely to the consequent increase in porous properties (pore volume and surface area) of the activated carbon samples. At the longest activation time of 240 min, the isotherms for both carbons changed from Type I to Type II with small hysteresis loops, indicating the presence of some mesopores. It was also noted that for the same activation time, the amount of N_2_ adsorbed with the A900 series was slightly higher than those of the A850 series because the former was prepared at a higher activation temperature, thus resulting in a larger extent of pore development due to the increase in char burn-off.

Activated carbon derived from the OTA method showed a distinct change in the isotherms from Type I for A850-60 and A900-60 to Type IV isotherm (Type II plus a hysteresis loop), with an increase in both the amount adsorbed and the size of the hysteresis loop as the number of preparation cycles was increased from cycle 1 to cycle 3. This indicates the progressive development of mesopores with the distribution of pore sizes. The hysteresis loop closed at the relative pressure of about 0.4 for all samples and its shape resembled Type H2 for the classification of hysteresis loops, according to the IUPAC classification [29], which indicated that the adsorbent consists of interconnected networks of pores of different sizes and shapes [30].

It is further noted from N_2_ isotherms in Figure 2 that sample A850-60-3 had greater amounts of N_2_ adsorbed as compared to that of sample A900-60-3, although the latter was activated at a higher temperature. This can be explained based on the porous properties of activated carbon presenting in the next section, as shown in Table 3. It was discovered that the mesopore volume, the total pore volume, and surface area of A900-60-3 were slightly lower than those of sample A850-60-3, thus allowing a lower amount of N_2_ to be adsorbed on the carbon surface, as shown in Figure 2. It is likely that for such a very high carbon burn-off (93.7%) of sample A900-60-3, some adjacent mesopores collapsed and combined to form a larger mesopore, thus providing the reduction in mesopore volume and a smaller surface area.

### 2.4. Porous Properties of Prepared Activated Carbons

Table 3 compares the porous properties of activated carbons prepared by the two-step activation method and by the OTA method for the activation temperatures of 850 and 900 °C conditions. If we consider first the results of activated carbons produced by the two-step activation method for the A850 and A900 series (samples nos. 1-4 and 8-11), foreach carbon series, all porous properties including the average pore size, the BET surface area, the micropore volume, the mesopore volume, and the total pore volume increased with the increase in activation time from 60 to 240 min. This is ascribed to the increase in the carbon burn-off with increasing activation time that removes more carbon atoms via the CO_2_ gasification reaction. The percentage of micropore volume relative to the total pore volume dropped from 91.1 to 69.7% and from 86.0 to 67.0% for A850 and A900 carbon series, respectively, as the activation time was increased from 60 to 240 min. Obviously, employing a higher activation temperature of 900 °C promotes higher values of porous properties as compared to the lower temperature of 850 °C. An increase in the average pore size as the activation time was increased should result from the enlargement of some small pores caused by the effect of CO_2_ gasification, not by the pore coalescence which usually occurs at a relatively high carbon burn-off. The amount of mesopore volume increased from 0.024 to 0.178 cm^3^/g (8.9 to 30.3% of total pore volume) and from 0.041 to 0.270 cm^3^/g (14.0 to 33.0% of total pore volume) for A850 and A900 carbon series, respectively, as the activation time was increased from 60 to 240 min. From these results, it can be inferred that under the preparation conditions studied, the two-step activation with CO_2_ produced logan-seed activated carbon which mostly contains the micropore size range.

Next, the porous properties of activated carbon produced by the OTA method are considered. Again, increasing the activation time gave rise to the increase in all porous properties for the A850 series (sample nos. 1, 5–7) and A900 series (sample nos. 8, 12–14). It was noticed that activated carbons from the OTA preparation showed remarkably higher volumes of both micropores and mesopores for the same activation time as compared to those obtained from the two-step activation method (for example, comparing sample no. 3 vs. no. 6 and no. 11 vs. no. 14). The mesopore volume and the total surface area also increased with the increase in the number of repeated cycles of the OTA method (sample nos. 5–7 and sample nos. 12–14). The maximum amount of mesopores of 0.474 cm^3^/g, corresponding to 44.1% of the total pore volume, and maximum BET surface area of 1773 m^2^/g was achieved for sample no. 7 (A850-60-3) for the three-cycle treatment of the OTA preparation method. It was noted that the activated carbon produced from the three-cycle preparation of the OTA method at the higher activation temperature of 900 °C (sample no. 14, A900-60-3) showed a slight decrease in porous properties, as compared to sample no. 7 (A850-60-3) of lower activation temperature. This is probably caused by the merging of a number of mesopores of sample A900-60-3, resulting in an increase in the average pore size from 2.42 nm for A850-60-3 to 2.54 nm for A900-60-3. For comparison, the activated carbon prepared by the two-step activation (sample no. 11, A900-240) provided the maximum amount of mesopores and surface area of 0.270 cm^3^/g and 1499 m^2^/g, respectively, using the activation temperature and time of 900 °C and 240 min, respectively. It is therefore obvious that the OTA method is an effective means for producing activated carbon from longan seed with increasing amounts of mesopores and a high surface area, as compared to the two-step activation method with a comparable carbon burn-off.

Table 4 shows the values of the BET surface area and mesopore volume of activated carbons produced by various activation techniques from previous investigations, with the purpose of increasing the amount of mesopores. These results show that chemical activation and chemical activation augmented with physical activation are quite effective in increasing the mesopore volume in the derived activated carbon, except for the activation with NaOH, which gives relatively low values of surface area and the amount of mesopores. The OTA preparation method proposed in this study provided activated carbon with a reasonable amount of developed mesopores and a high specific surface area. More importantly, it causes less pollution and involves a lower preparation cost, as compared to the traditional chemical activation methods.

Obviously, the extent of pore development is clearly dependent on the time and temperature of activation which in turn have a direct bearing on the carbon burn-off during char activation, where the percentage of carbon burn-off during the activation step is determined by the following equation,

(1) $$Burn-off(\%)=\frac{W_{char}-\;W_{AC}}{W_{char}}\;x\;100$$

where *W_char_* and *W_AC_* are the initial weight of char and the weight of activated carbon, respectively.

**Figure 3:**
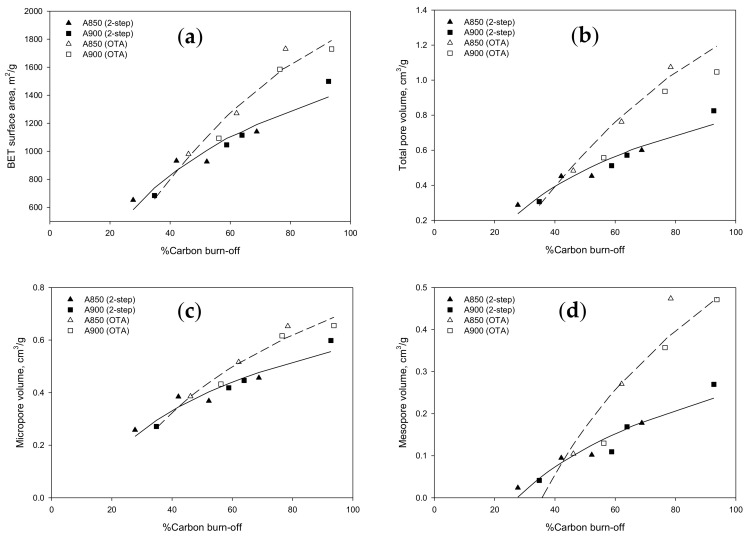
Effect of carbon burn-off during CO_2_ activation step of longan seed biomass on the porous properties of activated carbons prepared by the two-step activation and the OTA methods, (**a**) BET surface area, (**b**) total pore volume, (**c**) micropore volume, and (**d**) mesopore volume.

Therefore, it is possible that the effect of time and temperature of activation could be consolidated into a single effect of char burn-off on the porous properties of the prepared activated carbons. Figure 3 typically compares the dependence of BET surface, total pore volume, micropore volume, and mesopore volume on the percentage of char burn-off of activated carbons prepared by the two-step activation and the OTA methods, respectively. It is clear that the porous properties of activated carbon correlated well with the percentage of char burn-off for each preparation method and the porous properties of activated carbons tended to increase with the increase in % char burn-off, as expected. It is interesting to observe further that the porous properties of activated carbons produced by the OTA method appeared to increase with increasing char burn-off at a much faster rate (steeper slope of the curve). These results suggest that for the same char burn-off level the OTA method was able to significantly increase the char reactivity for CO_2_ gasification over that of activated carbon produced by the conventional two-step activation method. To correlate the porous properties of prepared activated carbon (y) as a function of the percentage of char burn-off (x), empirical equations were proposed and presented in Table 5, along with the regression coefficient of the fitting (R^2^). Overall, the proposed equations are capable of predicting the porous properties of the prepared activated carbon with reasonable accuracy.

The correlation between the percentage of carbon burn-off and porous properties of the prepared activated carbon by CO_2_ activation present in Figure 3 were also found in a number of previous investigations using different types of precursors, for example sewage sludge [31], waste tires [32], and sugarcane bagasse [33]. It was summarized that the porous properties of activated carbon, including the micropore volume, the total pore volume, and BET surface area, tended to increase almost linearly with the increase in activated carbon burn-off to the value of about 70% and then decreased at higher burn-off. The reduction in porous properties at a high carbon burn-off was explained to result from the coalescence of adjacent small pores to form larger-size pores with lower surface area and chemical reactivity. However, as shown in Figure 3, the porous properties of activated carbon prepared by the two-step activation in the present study showed a continuous increase in porous properties up to a very high degree of char burn-off of 92.7%, although a slight drop in porous properties was observed for activated carbon produced by the OTA method over the burn-off values from 78.4 to 93.7%. It is believed that all porous properties will finally decrease when almost all carbon atoms are consumed by the CO_2_ gasification. The different results could depend to a larger extent on the difference in the chemical nature of the precursor used and to a lesser extent on the preparation conditions employed for activated carbon synthesis.

### 2.5. On Mesopore Development by the OTA Method

The porous properties of activated carbon presented in the previous section indicated that the incorporation of oxidation and thermal destruction steps for initial activated carbon with a certain degree of carbon burn-off prior to the subsequent activation step, referred to as the OTA preparation method, was able to effectively promote the development of mesopores in longan seed-based activated carbon. In addition to producing a higher amount of mesopores as compared to the two-step activation method, the OTA method was also capable of increasing the amount of micropores under the same activation conditions of time and temperature, which also produced a higher total BET surface area.

**Figure 4:**
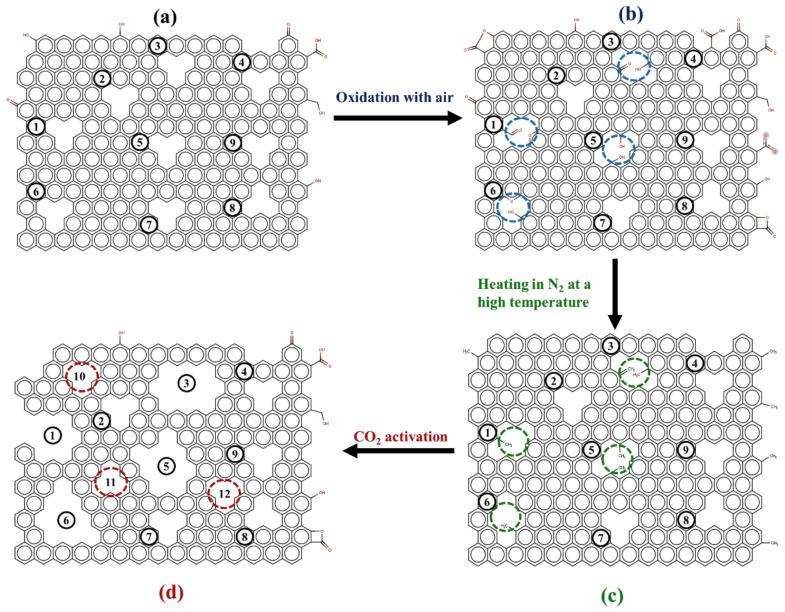
The model for mesopore development by the OTA method is represented schematically by the following steps, (**a**) the initial activated carbon contains a certain number of micropores (nos. 1–9), (**b**) formation of oxygen functional groups on the surface of some original micropores (nos.1, 3, 5, 6) by air oxidation, (**c**) bond dissociation of the functional groups increases the surface reactivity of micropores (nos.1, 3, 5, 6) due to the increasing number of free radicals and unpaired electrons, and (**d**) formation of new mesopores (nos.1, 3, 5, 6) by enlargement as well as the formation new micropores (nos. 10–12) by CO_2_ gasification reaction.

Based on the porous properties results of the prepared activated carbon in Table 3, the mechanism of mesopore development was proposed in this study, which is schematically presented in Figure 4, starting from Figure 4a, which represents an initial activated carbon prepared by the two-step activation method, for example, A850-60 carbon, which contains a certain amount of micropores (pore nos. 1–9). Next, some micropores (labeled as pore nos. 1, 3, 5, and 6) containing hetero atoms which are reactive enough to be oxidized by oxygen in the air form a certain quantity of oxygen functional groups on the surfaces of pore nos. 1, 3, 5, and 6, as shown in Figure 4b. After heating the carbon in an inert atmosphere of nitrogen at a high temperature, the bonds of the surface functional groups are destroyed, creating more surface defects with a high degree of surface reactivity due to the presence of a large number of unpaired electrons and free radicals, as illustrated in Figure 4c. The number of reactive pores subjected to functional group formation will depend on the distribution of the activation energy level for bond formation, which in turn is dictated by the oxidation temperature employed. Finally, the resulting carbon is further activated in carbon dioxide, as shown in Figure 4d. This produces the enlargement of the original reactive pores (nos. 1, 3, 5, and 6) by the gasification reaction, thus creating larger size mesopores as well as some newly formed micropores (nos. 10, 11, and 12). Repeating the OTA cycle will increase the amount of mesopores and additional micropores, but mesopores are developed in a greater proportion due to the higher degree of surface reactivity towards CO_2_ gasification.

To support the proposed mechanism of mesopore development by the OTA method, experimental evidence is presented in the following sections for the increased amount of surface functional groups after air oxidation, the increase in carbon reactivity towards CO_2_ gasification after the heat treatment step, and the pore size distributions of the mesoporous activated carbon after the reactivation step.

### 2.6. Surface Functional Groups of Prepared Activated Carbon

**Figure 5:**
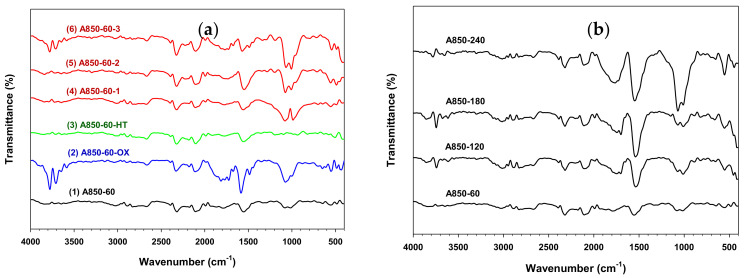
The FTIR spectra of the derived activated carbon prepared by (**a**) the OTA method, including (1) the initially activated carbon (A850-60), (2) the air-oxidized carbon (A850-60-OX), (3) the heat treatment carbon (A850-60-HT), (4) the reactivated carbon (A850-60-1), (5) A850-60-2, and (6) A850-60-3, and (**b**) by the two-step activation method for varying activation time.

The FTIR results of activated carbon samples from the OTA method, namely the original carbon from the two-step activation (A850-60), the air oxidized sample (A850-60-OX), a sample with heat treatment (A850-60-HT), and the final reactivated carbons from cycles 1–3 (A850-60-1, A850-60-2, and A850-60-3) are shown in Figure 5a. For the initially activated carbon (sample no. 1, A850-60), a peak detected near the wavenumber of 2100 cm^−1^ is assigned to terminal alkyne [34], while the peak near 2320 cm^−1^ could be attributed to NH stretching vibration [35], or carbon-oxygen groups due to ketone [36]. A peak detected at 1554 cm^−1^ is generally assigned to ring vibrations in large condensed aromatic carbon skeletons or likely responding to aromatic rings [37,38]. These aromatic rings are usually associated with the appearance of weak to medium absorption around 3000 to 3150 cm^−1^. Two small peaks at 1080 and 1016 cm^−1^ are due to the vibration of C-O stretching of primary alcohols, and O-H bending modes of phenolic, alcoholic, and carboxylic groups [36,38]. For air-oxidized carbon (sample 2, A850-60-OX), there exist two sharp peaks in the range of 3600–3780 cm^−1^ which have been assigned to isolated hydroxyl groups (O-H) stretching [39]. There is also another large peak between 1730 and 1880 cm^−1^, which indicates strong carbonyl double bonds (C=O) such as anhydrides, aldehydes, carboxyl, and lactones [40,41]. It is obvious that the oxidation of activated carbon with air could introduce a number of surface functional groups on the graphene sheet of activated carbon.

After heating the oxidized carbon at a high temperature in nitrogen, the heat-treated sample (A850-60-HT) showed several peaks at 2329, 2107, and 1557 cm^−1^, which are the same as the initial activated carbon. However, no peaks were detected over the range 1100–1000 cm^−1^, which indicates the removal of oxygen functional groups such as phenolic and carboxylic groups as a result of the heat treatment. The reactivated carbons by CO_2_ (sample nos. 4–6 for A850-60-1, A850-60-2, and A850-60-3) showed similar spectra to the initial activated carbon but with significant bands between 1100 and 1000 cm^−1^, which is indicative of functional group formation by CO_2_ oxidation during the activation step. The similar FTIR spectra pattern of the three activated carbons indicated that the number of the repeated cycle for producing mesoporous activated carbon by the OTA method had no influence on the types of the created surface functional groups.

The FTIR results of four activated carbon samples produced from the two-step activation method are shown in Figure 5b. It is observed that increasing the activation time from 60 to 240 min had virtually no bearing on the FTIR spectra pattern and the detected peaks were similar to those of activated carbons produced by the OTA method. It is further observed that a sharp peak was detected at 1551 cm^−1^, and another large peak was detected between 1730 and 1880 cm^−1^. In addition, the peak intensity of C-O stretching and O-H bending at 1072 and 1016 cm^−1^ for A850-240 was higher than those of the other samples, implying that the amounts of oxygen-containing functional groups can be affected by varying the activation time. This is in agreement with studies from the previous investigation [42].

On Boehm’s titration results, Table 6 typically shows the amounts of oxygen functional groups for activated carbon samples as basic and acid groups for the OTA preparation method. The surface chemistry of activated carbon is related to the presence of heteroatoms within the carbon matrix that can form various oxygen surface groups during the activation step by oxidizing gases such as oxygen, carbon dioxide, and steam or by direct oxidation in the liquid or gas phase [43]. The presence of oxygen functional groups renders activated carbon an acid-base character. Examples of acid functional groups are carboxylic, phenolic, and lactonic groups, whereas carbonyl and chromene groups are the typical basic groups. Boehm’s titration results showed that after A850-60 was oxidized with air, the amount of acidic groups increased substantially by about five times from 0.069 to 0.393 mmol/g for sample A850-60-OX, while the amount of basic groups decreased by about 18% from 1.090 to 0.894 mmol/g. After heating the oxidized carbon in the inert atmosphere of N_2_ at a high temperature, almost all of the acidic groups were removed, with a remaining amount of about 0.002 mmol/g, whereas heat treatment increased the amount of basic groups from 0.894 to 1.331 mmol/g (sample A850-60-HT). Interestingly, the thermal destruction of oxygen functional groups from the surface of activated carbon can be used as one of the methods to increase the concentration of the basic functional groups on the carbon surface. The increase in the basicity of carbon surface enhances the capture of acidic gases such as carbon dioxide and hydrogen sulfide [44,45,46]. The reoxidation of activated carbon in cycle 2 (sample A850-60-1-OX) led to an increase in acidic groups which was twice as high, from 0.152 to 0.467 mmol/g, as compared to activated carbon from cycle 1 (A850-60-1), while the basic groups dropped slightly from 1.295 to 1.144 mmol/g. It can be said that there is an agreement in the qualitative and quantitative variations of the surface functional groups of activated carbon that went through the OTA preparation method, as analyzed by both the FTIR and the Boehm titration technique.

### 2.7. Carbon Reactivity towards CO_2_ Gasification

**Figure 6:**
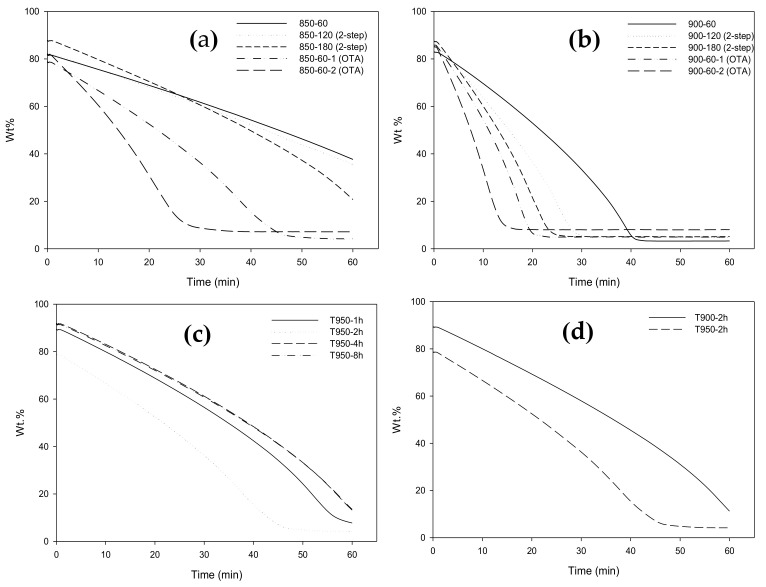
TG curves for CO_2_ gasification of longan-seed activated carbons prepared by the two-step activation and the OTA methods for activation temperatures of (**a**) 850 °C and (**b**) 900 °C, and TG curves for A850-60 carbon showing, (**c**) the effect of heat treatment time at temperature of 950 °C and (**d**) the effect of heat treatment temperature for the treatment time of 2 h.

The reactivity of activated carbon for CO_2_ gasification was determined by following the weight loss of a carbon sample as a function of time in the thermogravimetric analyzer under a constant flow of carbon dioxide. Figure 6a,b shows the TGA data of the time-residual weight of activated carbon samples prepared via the OTA method and the two-step activation method at two activation temperatures of 850 and 900 °C, respectively. For a clearer explanation, the meanings of labels in the figures are typically shown below for the gasification temperature of 850 °C in the TGA.

850-60 for weight loss data of the prepared char in TGA in CO_2_ at 850 °C for 60 min

850-120 (two-step) for weight loss data of activated carbon, prepared in a tube furnace in CO_2_ at 850 °C and 60 min, in TGA at 850 °C for 60 min

850-180 (two-step) for weight loss data of activated carbon, prepared in a tube furnace in CO_2_ at 850 °C and 120 min, in TGA for 60 min

850-60-1(OTA) for weight loss data of heat-treated carbon, prepared in tube furnace from cycle 1, in TGA for 60 min

850-60-2 (OTA) for weight loss data of heat-treated carbon, prepared in tube furnace from cycle 2, in TGA for 60 min

Based on the derived TGA data, the fractional char conversion (*α*) for gasification with CO_2_ in a thermogravimetric analyzer was calculated based on the following defining equation.

(2) $$\alpha =\frac{\left(W_{0}-W\right)}{\left(W_{0}-W_{ash}\right)}$$

where *W*_0_, *W* and *W_ash_* are the initial weight of char before gasification, the weight of char at time t, and the weight of ash in the char sample, respectively. The reactivity of activated carbon for CO_2_ gasification (*R_c_*) is defined as the rate of change of the fractional char conversion as follows.

(3) $$R_{c}=\frac{d\alpha }{dt}=-\frac{\left(d\alpha /dt\right)}{\left(W_{0}-W_{ash}\right)}$$

**Figure 7:**
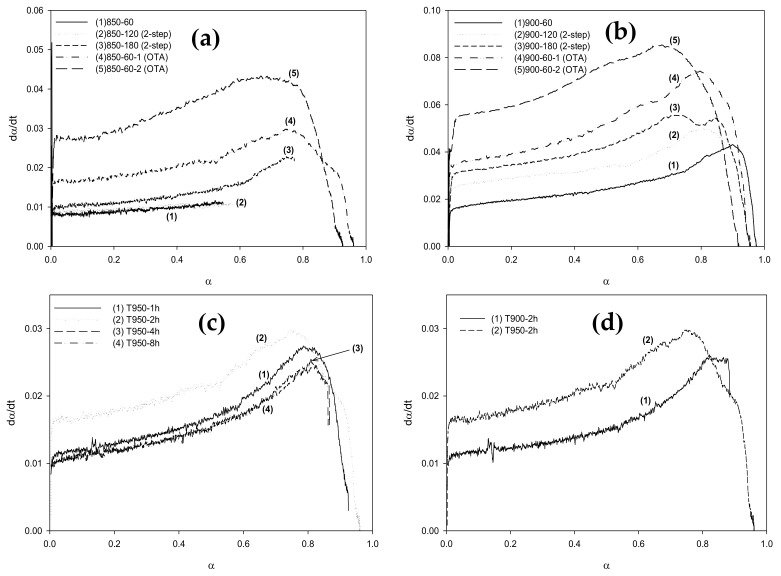
Effect of carbon conversion (**a**) on char reactivity (R_c_) towards CO_2_ gasification of Table 850. °C and (**b**) 900 °C, and the effect of (**c**) heat treatment time and (**d**) heat treatment temperature on the char reactivity.

The carbon reactivity (R_c_) was calculated from the TGA data as a function of the fractional conversion (α) using the numerical discrete method, that is, R_c_ = −(∆W/∆t)/(W_0_−W_ash_), and the calculated results are shown in Figure 7a,b. The reactivities of all samples show a characteristic continuous increase with the increase in fractional conversion, which passed through a maximum at a certain fractional char conversion, depending on the given type of activated carbon. The rising trend of reactivity curves could possibly be attributed to the increase in surface area with increasing carbon conversion that provided more active sites for the gasification, leading to a higher reaction rate. The falling of the reactivities at a high fractional carbon conversion of around 0.80 or at the carbon burn-off of 80% is probably due to the consolidation of some adjacent pores that results in larger average pore sizes that produce a decrease in the surface area and hence a reduction in the gasification rate. It should be noted also that the carbon reactivity for the gasification at 900 °C is about twofold higher than that at 850 °C, thus indicating a strong effect of the gasification temperature on the carbon reactivity.

At the same carbon conversion (α), the reactivity of carbon appeared to increase for activated carbons prepared at a longer activation time for the two-step activation method, as observed from the comparison of the curves of symbols (1), (2), and (3) in Figure 7a,b. When the fractional carbon conversion was smaller than 0.80, the reactivity of carbon obtained from cycle 2 was higher than that of cycle 1 for the OTA preparation method. It was also noted that the reactivity of activated carbon from the OTA method was higher than that derived from the two-step activation method at the same activation time and temperature, for example, if we compare condition (2) with (4) and (3) with (5). These results indicate that the increase in the surface area as the carbon conversion increases brings about an increase in the number of active sites for the gasification reaction by carbon dioxide.

The effects of oxidation time and heat treatment temperature of the OTA method on the TGA weight loss and carbon reactivity are also shown in Figure 6c,d, and Figure 7c,d, respectively, for activated carbon prepared at the activation temperature of 850 °C. For the oxidation temperature of 950 °C, the carbon reactivity increased with an increase in the oxidation time only up to the period of 4 h and the increase in oxidation temperature from 900 to 950 °C enhanced the carbon reactivity by about 50%. Therefore, it is expected that the development of mesopores and surface area by the OTA method can also be affected by the time and temperature used during the oxidation step.

### 2.8. Pore Size Distributions and Surface Morphology of Prepared Activated Carbons

**Figure 8:**
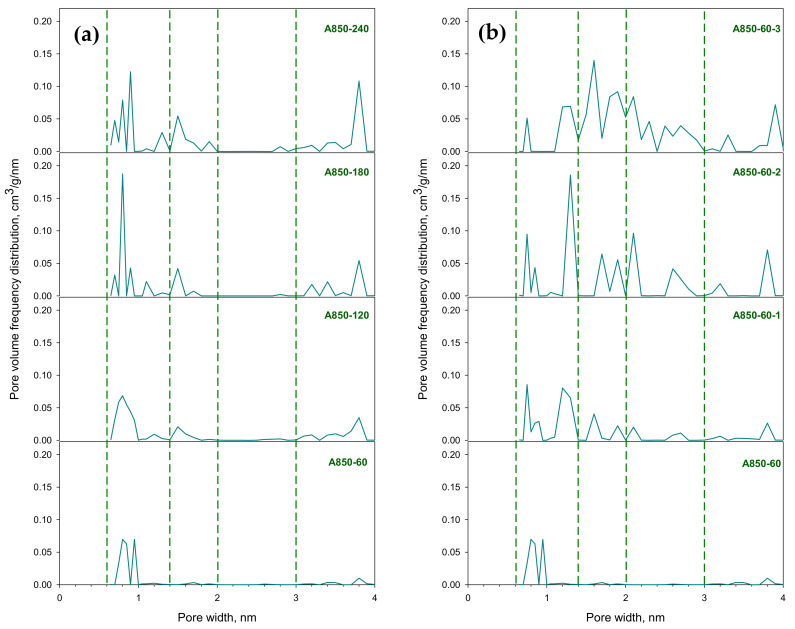
Pore size distributions of longan seed-derived activated carbon prepared by (**a**) the two-step activation method and (**b**) the OTA method, as determined by the GCMC simulation.

Figure 8 shows the typical variations of pore size distributions of activated carbon samples prepared by the two-step activation method (A850 carbon series) as a function of activation time and the OTA method as a function of a repeated preparation cycle. For the sake of discussion, the pore size distributions are divided into five ranges: <0.6 nm (ultra-micropores), 0.6–1.4 nm (micropores), 1.4–2 nm (super-micropores) and 2–3 nm, and 3–4 nm for the mesopore size ranges. Table 7 shows the calculated pore volume data for each pore size range. The initial activated carbon (A850-60) contains mainly micropores (0.6–1.4 nm) and a small amount of mesopores in the range of 3–4 nm. After further activation by the two-step activation method at various times, the derived activated carbons show a multi-modal pore size distribution. By activating the initial activated carbon in CO_2_ for 60 min, the volume of micropores (0.6–1.4 nm) increased by 26.4% from 0.242 to 0.306 cm^3^/g. However, a further increase in the activation time from 60 to 240 min had no significant effect on the micropore volumes. On the other hand, the volume of super-micropores (1.4–2 nm) tended to increase continuously from 0.006 to 0.102 cm^3^/g, as the activation time was increased from 60 to 240 min. The mesopores were created in the upper pore size range of 3–4 nm and increased substantially from 0.022 to 0.166 cm^3^/g, as the activation time was increased from 60 to 240 min.

For mesoporous activated carbon produced by the OTA method, it is seen that the total pore volume increased from 0.480 to 1.074 cm^3^/g, which is an increase of about 124% when using three preparation cycles from cycles 1 to 3. The pore size distribution of all three samples (A850-60-1, A850-60-2, and A850-60-3) covered every pore size range from 0.6–4 nm. In general, the shape of the distribution curve for each pore size is quite similar except that the size of the peak or magnitude of the area under the curve is different. The volume of micropores in the size range from 0.6 to 1.4 nm seemed to increase from 0.309 for cycle 1 to 0.339 cm^3^/g for cycle 2 and then dropped to the value of 0.207 cm^3^/g for the cycle 3 sample. This could have resulted from the transformation of some micropores to larger super-micropores (1.4–2 nm) as a result of the gasification reaction. The substantial increase in the volume of super-micropores from the values of 0.066 to 0.393 cm^3^/g at the expense of smaller micropores was obvious when the three OTA preparation cycles were used. The progressive increases in the volume of the mesopores in the range of 2–3 and 3–4 nm are discernible in Figure 8, with a pore size range from 2 to 3 nm constituting about 43.8, 65, and 70% of the total mesopore volume for carbon samples produced from cycle 1, cycle 2 and cycle 3 of the OTA method, respectively. This indicates a significant increase in the chemical reactivity of activated carbon with an increasing number of repeated cycles of the OTA method.

**Figure 9:**
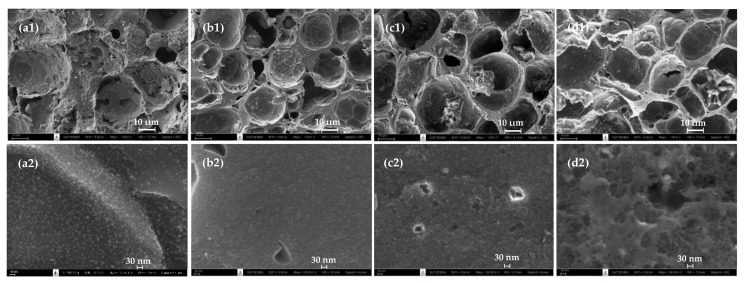
SEM micrographs of activated carbon prepared by the OTA method for (**a**) A850-60, (**b**) A850- 60-1, (**c**) A850-60-2, and (**d**) A850-60-3, with magnification of 1K for (**a1**–**d1**) and 100K. for (**a2**–**d2**).

Figure 9 shows the surface images obtained from a field emission scanning electron microscope (FE-SEM, Carl Zeiss, Model Auriga) of mesoporous activated carbon prepared by the OTA method for the original carbon (A850-60) and activated carbons from cycle 1 (A850-60-1), cycle 2 (A850-60-2) and cycle 3 (A850-60-3). Figures a1, b1, c1, and d1 and a2, b2, c2, and d2 present low and high magnifications, respectively. It can be seen that there was a tendency for an increase in the average pore size and the number of pores with increases in the number of treatment cycle of the OTA method, although the change is not very pronounced. These results are in agreement with those of the average pore size and pore volume results as presented in Table 3. 

In summary, the OTA method is a modified method of the conventional two-step activation method for producing mesoporous activated carbon by incorporating two consecutive steps of air oxidation and thermal destruction of the developed surface functional groups prior to activation by CO_2_ oxidizing gas. Mesopores are created not by the coalescence of micropores at a high degree of carbon burn-off, but as a result of micropore widening caused by the increased surface reactivity of micropores for CO_2_ gasification. Therefore, with the OTA method, the creation of mesopores can occur at any level of char burn-off.

## 3. Materials and Methods

### 3.1. Raw Materials

Longan seed was used as the raw material in this work for the preparation of activated carbon. It was supplied by Saha-Prachinburi Foods Industry Ltd., a local fruit processing plant in Chiangmai province, Thailand. Longan seed is a round black inner seed of longan fruit and has a diameter of around 1 cm. N_2_ gas of high purity grade (99.99%), CO_2_ gas of high purity grade (99.99%), and air (99.99%) were supplied in gas cylinders by Linde (Thailand) PCL. The longan seed sample was kept for char preparation and was characterized for proximate analysis (wt% fixed carbon, volatile content, and ash content) and ultimate analysis (wt.% C, H, N, S, and O). The proximate analysis was performed using a thermogravimetric analyzer (TGA/DSC 1 STAR^e^ System, METTLER TOLEDO, Switzerland) and the heating scheme proposed by Guo and Lua [47]. The ultimate analysis was determined by a CHNS analyzer (CHN 628S, LECO Corporation, USA). The thermal decomposition behavior of the longan seed precursor was also studied using the TGA by non-isothermal heating from room temperature to the final temperature of 700 °C using a heating rate of 10 °C/min under a constant flow of nitrogen gas.

### 3.2. Preparation of Char and Microporous Activated Carbons

The as-received longan seed was rinsed and cleaned thoroughly with water and dried in an electric oven at 110 °C for 24 h. The dried longan seed was crushed in a jaw crusher and sieved to obtain an average particle size of 1.70 mm (10 × 14 US mesh designation). The char was prepared by loading about 50 g of the longan seed sample in a ceramic boat and was heated in a horizontal electric tube furnace of 7 cm inside diameter and 100 cm length (CTF 12, Carbolite, UK) from room temperature to 500 °C at the rate of 10 °C/min under a constant flow of nitrogen (100 cm^3^/min) and held at this temperature for 90 min. After that, the furnace was turned off and the char obtained was cooled down to room temperature inside the furnace under the flow of nitrogen. These carbonization conditions for char preparation were employed to ensure a complete devolatilization of the raw biomass. Next, about 15 g of the prepared char were gasified (activated) in a quartz tube reactor of 3.0 cm inside diameter and 120 cm length inserted in a vertical tube furnace (Carbolite, UK) under a constant flow of CO_2_. The activation temperatures used were 850 and 900 °C and the holding time was varied between 60 and 240 min, giving carbon burn-off for the activation step of up to 90%.

### 3.3. Preparation of Mesoporous Activated Carbon by the OTA Method

The OTA method for preparing mesoporous activated carbon from longan seed was carried out in the same quartz tube reactor inserted in the vertical tube furnace (CTF 12, Carbolite, UK). About 15 g of the activated carbon prepared in Section 3.2 with the carbon burn-off of around 30% was oxidized in the reactor by heating the activated carbon from room temperature in a stream of air (100 cm^3^/min) to the oxidation temperature of 230 °C and held at this temperature for 12 h. The purpose of this step was to create surface functional groups particularly at the newly formed pores of the original activated carbon. Next, the carbon sample was heated at 950 °C for 2 h under nitrogen at a flow rate of 100 cm^3^/min to remove the surface functional groups, leading to the formation of more active sites for further gasification reactions at the originally formed micropores. After that, the sample was gasified with CO_2_ again at the desired activation temperature of 850 or 900 °C for 60 min to enlarge the original micropores, thus producing an increasing amount of mesopore volume. This process completed the first cycle (cycle 1) of the OTA preparation procedure and a total of three cycles (cycles 1, 2, and 3) were performed, using the same activation time (60 min) and temperature (850 or 900 °C) for each cycle. The derived microporous carbon prepared in Section 3.2 using the two-step activation was designated as AX-Y, where A stands for activated carbon and symbols X and Y represent the activation temperature (850 or 900 °C) and activation time (60, 120, 180, or 240 min), respectively. The mesoporous activated carbon prepared by the OTA method was designated as AX-Y-Z, where A stands for activated carbon originally prepared by the two-step activation method at the activation temperature X (850 or 900 °C) and time Y (60 min), and Z is the number of repeated cycles (1, 2 or 3) for the processes of oxidation, heat treatment and reactivation at the same temperature X and time Y. The final yield of activated carbon was calculated based on the weight of the original longan seed char.

Activated carbons prepared by the two-step activation and the OTA method were also analyzed for the proximate and ultimate analyses.

### 3.4. Porous Properties of the Prepared Activated Carbons

Porous properties of the derived activated carbons were determined from the adsorption/desorption isotherms of nitrogen at −196 °C measured by using an Accelerated Surface Area and Porosimetry Analyzer (ASAP2010, Micromeritics, USA). The specific surface area was calculated from the N_2_ adsorption isotherm data for relative pressures (P/P^0^) over a range from 0.001 to 0.25 by applying the Brunauer–Emmett–Teller (BET) equation [48]. The pore size distribution data generated from GCMC simulation using the N_2_ adsorption isotherms (see Section 3.7 for the methodology of GCMC simulation) were used to compute the micropore volume and total pore volume which were taken as the cumulative pore volumes for pore sizes smaller than 2 and 4 nm, respectively. Alternatively, they can be estimated from the areas under the curves of pore size distributions as shown in Figure 8. The mesopore volume was derived by subtracting the micropore volume from the total pore volume. The average pore size was computed based on the equation 4V_T_/A, assuming cylindrical pores, where V_T_ is the total pore volume and A is the BET surface area.

### 3.5. Surface Reactivity of Activated Carbon towards CO_2_ Gasification Reaction

To assist the understanding of pore development in activated carbon prepared by the OTA method, the reactivity of activated carbon towards CO_2_ gasification reaction was studied by following the weight loss of a sample in a thermogravimetric analyzer (TGA/DSC 1 STAR^e^ System, METTLER TOLEDO, Switzerland). About 15 mg of the initial activated carbon sample, prepared from activating char in CO_2_ at 850 and 900 °C, which went through the oxidation and heat treatment in the tube furnace was loaded into an alumina crucible (70 µL capacity) of the thermogravimetric analyzer and heated from room temperature to the desired gasification temperature at the heating rate of 20 °C/min under a constant flow of nitrogen at 100 cm^3^/min. When the final gasification temperature was reached, the gas flow was switched from nitrogen to carbon dioxide flowing at the rate of 100 cm^3^/min and the sample was held at this temperature for 60 min. The sample weight loss via gasification reaction was continuously recorded as a function of time. The gasification (activation) temperatures of 850 and 900 °C were studied for the activated carbons after being oxidized and heat-treated in the tube furnace for cycles 1 and 2 of the OTA preparation method. For comparison, the TGA measurement was also carried out on longan seed char for CO_2_ gasification at 850 and 900 °C as a function of time to determine its carbon reactivity for the two-step activation method.

### 3.6. Surface Functional Groups of Activated Carbons

The presence of surface functional groups upon air oxidation of activated carbon was determined qualitatively by Fourier transform infrared spectroscopy (FTIR) and quantitatively by applying the Boehm titration technique [49,50,51,52]. For the FTIR analysis, about 0.50 g of an activated carbon powder was placed in a micro-sample holder of an FTIR spectrophotometer (Vertex 70 FT-IR, Bruker, USA). The IR spectra were collected by the acquisition of 64 scans for each run, using the wavenumber in the mid-IR spectrum (400–4000 cm^−1^) with an incremental measurement resolution of 4 cm^−1^. For the Boehm titration analysis, 0.50 g of the carbon sample was loaded into two Erlenmeyer flasks, each of which was filled with 25 mL of 0.05 M solutions of sodium hydroxide and hydrochloric acid. Next, each flask was sealed and shaken at room temperature for 36 h, and after that, the mixture was filtered using Whatman 44 filter paper. Five milliliters of the filtrate from each flask were pipetted and the excess base and acid remaining in the filtrate were determined by titrating with hydrochloric and sodium hydroxide solutions, respectively. The amounts of total acid groups were determined under the assumption that NaOH neutralizes the carboxylic, lactonic, and phenolic acid groups. The number of basic groups was determined from the amount of hydrochloric acid that reacts with the basic surface groups on the carbon surfaces. Dried potassium hydrogen phthalate (KHP) was the primary standard used to standardize NaOH and HCl concentrations for the correct calculation of the amounts of surface oxygen functional groups. Titration of the sample solution was performed in triplicate and the average value was used for determining the amounts of surface functional groups.

### 3.7. GCMC Simulation for Determining Pore Size Distributions in Activated Carbon

In this study, the GCMC simulation method proposed by Ravikovitch et al. [53] combined with the N_2_ adsorption isotherm data were used to calculate pore size distributions of the prepared activated carbons. For the simulation computation, N_2_ is modeled as a simple spherical Lennard-Jones (LJ) molecule and the molecular model of activated carbon is assumed to be a parallel pair of finite-length graphene layers. The interaction energy between two LJ sites is calculated using the Lennard–Jones 12-6 equation [54]. The LJ parameters are shown in Table 8. The simulation box for this ensemble is a finite length carbon slit pore and has a linear dimension of 6 nm in the x and y directions. We assumed that the top and the bottom of the simulation box are two walls of the slit pore, and each wall consists of three graphene layers. These layers are stacked on top of each other with an interlayer spacing of 0.3354 nm. The width H of this slit pore model is defined as the distance between a plane passing through all carbon atom centers of the outermost layer of one wall and the corresponding plane of the other wall. To simulate the N_2_ adsorption, the pore widths (H) are varied from 0.65 to 4.0 nm, to cover the micropore and mesopore size range in activated carbons. Each adsorption isotherm consists of 50 pressure points to cover the range of relative pressure (P/P^0^) from 1 × 10^−4^ to 1 and 40 local isotherms are then calculated for N_2_ at 77 K. The procedure for GCMC computation is as follows. We first specify the volume of the box, the chemical potential, and the temperature of the system to compute the adsorption equilibrium. One GCMC cycle consists of one thousand displacement moves and attempts of either insertion or deletion with equal probability. For an adsorption branch of the isotherm, 30,000 cycles are typically needed for the system to reach equilibrium, and additional 30,000 cycles are used to obtain the ensemble averages. For each point on the adsorption branch, we use an empty box as the initial configuration, and the simulation is carried out until the number of particles in the box no longer changes. The pressure of the bulk gas corresponding to a given chemical potential is calculated from the equation of state proposed by Johnson et al. [55]. For calculation of the PSD, the experimental N_2_ isotherms of activated carbon and the local isotherms computed by the simulation are then matched by using a solver optimization function of the EXCEL program to minimize the mean square error between experimental isotherms and the simulated isotherms.

## 4. Conclusions

The results obtained from this study indicate that the preparation of activated carbon from longan seed biomass via the OTA method is a simple and effective means for producing and controlling the proportional volume of mesopores. One cycle of the OTA method consists of three consecutive steps of air oxidation to form surface oxygen functional groups, the thermal destruction of the functional groups to enhance the surface reactivity caused by the increasing number of defects, and the reactivation of activated carbon by CO_2_ that results in the widening of the original micropores as well as the creation of more new micropores. The maximum amount of both mesopores (0.474 cm^3^/g) and BET surface area (1773 m^2^/g) was achieved by using three cycles of the OTA preparation method at the activation temperature of 850 °C and 60 min activation time for each treatment cycle. By comparison, the conventional two-step activation method yielded the maximum mesopore volume and surface area of 0.270 cm^3^/g and 1499 m^2^/g, respectively, under the activation conditions of 900 °C and 240 min. In this study, three OTA cycles have been studied to affect mesopore development. To increase the flexibility of controlling the amount of mesopores, further investigation may involve studying the effects of different types of biomass precursors, the time and temperature of the air oxidation step, the number of repeated thermal destruction steps, the time and temperature of carbon activation step, and the initial porous texture of activated carbon. Activated carbon produced by the OTA method is particularly suitable for use in a liquid adsorption system since its mesoporous structure could help increase the diffusion rate of relatively large size adsorbate molecules to the adsorption sites. The utilization of OTA activated carbon would be advantageous because of fast kinetics due to the ability to control the mesopore volume to suit the nature of a given adsorbate and a high adsorption capacity (equilibrium) due to the large specific surface area of the produced activated carbon.

## Figures and Tables

**Table 1:** Proximate analysis of longan seed and some industrial biomass wastes.

Material	Proximate Analysis (Dry Basis) [wt.%]		
	Fixed Carbon	Volatile Matters	Ash
Longan seed (this work)	22.34	76.51	1.15
Longan seed ^1^	19.60	78.70	1.70
Oil palm shell ^2^	19.80	77.60	2.60
Coconut shell ^3^	19.40	79.20	0.60
Peanut shell ^3^	16.50	81.00	2.50
Eucalyptus sawdust ^3^	15.38	83.70	0.90
corn cob ^3^	14.60	83.00	2.40

Sources: ^1^ [27]; ^2^ [26]; ^3^ [28].

**Table 2:** Proximate and ultimate analysis results of longan seed, longan seed char, and activated carbons prepared by the two-step activation and the OTA method.

Samples	Proximate Analysis (Dry Basis) [wt.%]			Ultimate Analysis (Dry Basis) [wt.%]				
	Fixed Carbon	Volatile	Ash	C	H	N	S	O_diff._
Longan seed	23.34	76.51	1.15	47.56	6.55	1.09	0.03	44.76
C500-90	83.58	14.81	1.61	85.92	3.55	2.32	0.00	8.20
A850-60	86.25	11.57	2.18	91.91	0.77	2.38	0.00	4.93
A850-120	83.21	11.49	5.3	85.58	0.85	2.47	0.00	11.10
A850-180	83.02	11.45	5.53	81.16	0.98	2.48	0.00	15.38
A850-240	82.58	11.32	6.1	76.75	0.88	2.50	0.00	19.87
A850-60-1	83.40	10.93	5.67	84.00	0.63	2.16	0.00	13.21
A850-60-2	82.57	10.89	6.54	81.71	0.61	1.99	0.00	15.70
A850-60-3	80.08	10.38	9.54	76.60	0.44	1.90	0.00	21.06

**Table 3:** Porous properties of activated carbons prepared by the two-step activation and the OTA methods.

Sample	Sample	Avg.pore	V_Mic_.	V_Mes_.	V_T_	V_Mes_/V_mic_	S_BET_	% Carbon	Yield
no.	code	dia.(nm)	(cm^3^/g) (%)	(cm^3^/g) (%)	(cm^3^/g)	(%)	(m^2^/g)	Burn-off	(%)
1	A850-60	1.67	0.248 (91.1)	0.024 (8.9)	0.272	9.5	652	27.7	72.3
2	A850-120	1.88	0.343 (78.5)	0.094 (21.5)	0.437	27.6	932	42.1	57.9
3	A850-180	1.91	0.340 (76.9)	0.102 (23.1)	0.442	30.0	926	52.2	47.8
4	A850-240	2.06	0.409 (69.7)	0.178 (30.3)	0.587	43.5	1140	68.8	31.2
5	A850-60-1	1.89	0.375 (78.1)	0.105 (21.9)	0.480	27.8	980	46.1	53.9
6	A850-60-2	2.32	0.466 (63.3)	0.270 (36.7)	0.736	58.1	1271	62.1	37.9
7	A850-60-3	2.42	0.600 (55.9)	0.474 (44.1)	1.074	78.9	1773	78.4	21.6
8	A900-60	1.73	0.253 (86.0)	0.041 (14.0)	0.294	16.3	684	34.8	65.2
9	A900-120	1.96	0.376 (77.5)	0.109 (22.5)	0.485	29.1	1046	58.8	41.2
10	A900-180	2.16	0.406 (70.6)	0.169 (29.4)	0.575	41.6	1115	63.9	36.1
11	A900-240	2.30	0.547 (67.0)	0.270 (33.0)	0.817	49.3	1499	92.7	7.3
12	A900-60-1	2.06	0.434 (77.0)	0.130 (23.0)	0.564	29.9	1093	56.2	43.8
13	A900-60-2	2.49	0.583 (62.0)	0.357 (38.0)	0.940	61.3	1585	76.5	23.5
14	A900-60-3	2.54	0.576 (55.0)	0.471 (45.0)	1.047	81.8	1730	93.7	6.3

Notes: %Yield = 100—%Burn-off.

**Table 4:** Preparation of activated carbon by various methods for the purpose of mesopore production.

Precursor	Activation Method	Mesopore Volume, cm^3^/g	BET Surface Area	References
		(% Total Pore Volume)	(m^2^/g)	
Coconut shell	ZnCl_2_ + CO_2_	1.364 (71%)	2191	[21,22]
Waste tires	HCl + steam	1.620 (74%)	1119	[24]
Date stems	H_3_PO_4_	0.993 (95%)	1455	[6]
Rice husk	KOH	0.691 (46%)	2696	[15]
Chitosan flakes	NaOH	0.157 (62%)	318	[8]
Coconut leaves	H_3_PO_4_	1.276 (93%)	982	[9]
Longan seed	OTA method	0.474 (44%)	1773	This study

**Table 5:** Empirical equations correlating porous properties of activated carbon (y) prepared by the two-step activation and the OTA methods as a function of % carbon burn-off (x).

Porous Properties	Activation Method	Empirical Equations	R^2^
(a) BET surface area (m^2^/g)	2-step	y = 668.72ln(x) − 1638.7	0.9312
	OTA	y = 1157.5ln(x) − 3462.4	0.9582
(b) Total pore volume (cm^3^/g)	2-step	y = 0.4289ln(x) − 1.2032	0.9194
	OTA	y = 0.8556ln(x) − 2.7839	0.9432
(c) Micropore volume (cm^3^/g)	2-step	y = 0.2336ln(x) − 0.5562	0.9111
	OTA	y = 0.3642ln(x) − 1.0278	0.9457
(d) Mesopore volume (cm^3^/g)	2-step	y = 0.1951ln(x) − 0.6463	0.9151
	OTA	y = 0.4914ln(x) − 1.7561	0.8943

**Table 6:** Amounts of oxygen functional groups on activated carbon surfaces determined by the Boehm titration technique.

Sample	Sample	Basic Groups	Acidic Groups	Total Groups
Number	code	(mmol/g carbon)	(mmol/g carbon)	(mmol/g carbon)
1	A850-60	1.090	0.069	1.159
2	A850-60-OX	0.894	0.393	1.286
3	A850-60-HT	1.331	0.002	1.333
4	A850-60-1	1.295	0.152	1.447
5	A850-60-1-OX	1.144	0.467	1.611

**Table 7:** The pore volume for the pore size distribution of longan seed-derived activated carbon.

Sample No.	Sample Code	Pore Volume for Pore width (cm^3^/g)					Total Pore Volume (cm^3^/g)
		0–0.6 nm	0.6–1.4 nm	1.4–2 nm	2–3 nm	3–4 nm	
1	A850-60	0.000	0.242	0.006	0.002	0.022	0.272
2	A850-120	0.000	0.306	0.037	0.006	0.088	0.437
3	A850-180	0.000	0.291	0.049	0.002	0.100	0.442
4	A850-240	0.000	0.307	0.102	0.012	0.166	0.587
5	A850-60-1	0.000	0.309	0.066	0.046	0.059	0.480
6	A850-60-2	0.000	0.339	0.127	0.176	0.094	0.736
7	A850-60-3	0.000	0.207	0.393	0.330	0.144	1.074

**Table 8:** Molecular parameters used in the GCMC simulation.

Type	Interacting Site	Energy Well Depth (ε/k_B_), K	Collision Diameter (σ), nm
N_2_ (Spherical model)	N_2_	101.5	0.3615
Activated carbon	C	28.0	0.34

## Data Availability

The data presented in this study will be available upon request.
